# Mobile phone dependency and adolescent exercise participation: a CB-SEM and fsQCA study on the roles of self-control, time management, and health awareness

**DOI:** 10.3389/fpsyg.2025.1666004

**Published:** 2025-08-19

**Authors:** Quan Zhang, Cong Li, Jianxin Liu

**Affiliations:** ^1^Pai Chai University, Daejeon, Republic of Korea; ^2^Capital University of Physical Education and Sports, Beijing, China

**Keywords:** mobile phone dependency, adolescent exercise participation, self-control, time management, health awareness, CB-SEM, fsQCA

## Abstract

**Objective:**

The study examined how mobile phone dependency (MPD) is linked to adolescents’ engagement in structured exercise and whether this association operates through three theoretically derived mechanisms—self-control (SC), time management (TM), and health awareness (HA). A complementary configurational analysis explored alternative combinations of these factors that lead to high or low exercise participation.

**Methods:**

A cross-sectional survey was administered to 1,404 Chinese students in Grades 5–9 (49.6% girls; 51% rural). Standardized scales assessed MPD, SC, TM, HA, and adolescent exercise participation (AEP). Covariance-based structural equation modeling (CB-SEM) estimated direct and mediated effects; bias-corrected bootstrap confidence intervals tested indirect paths. Fuzzy-set qualitative comparative analysis (fsQCA) identified sufficient configurations of conditions for high and non-high AEP and assessed robustness across calibration thresholds.

**Results:**

The SEM model exhibited excellent fit (*χ*^2^/df = 2.723, CFI = 0.979, TLI = 0.975, SRMR = 0.036, RMSEA = 0.035, 90% CI [0.031, 0.039]). MPD showed a significant total effect on AEP (*β* = −0.61, *p* < 0.001), of which 83.5% was transmitted through the three hypothesized mediators. Among single mediators, HA accounted for the largest share of the indirect effect (*β* = −0.15), followed by SC (*β* = −0.12) and TM (*β* = −0.03). Three two-step and one three-step chained paths were also significant (*p* < 0.01). FsQCA revealed three sufficient configurations for high AEP: (a) low MPD + high SC + high HA, (b) low MPD + high TM + high HA, and (c) high SC + high TM + high HA irrespective of MPD. One configuration—high MPD combined with low SC, TM, and HA—was sufficient for non-high AEP. All solutions were robust to parameter changes.

**Conclusion:**

Mobile phone dependency undermines adolescent exercise primarily by eroding self-regulatory capacity, disrupting time structure, and diminishing health motivation. Nonetheless, strong personal resources can offset the risks of heavy phone use. Interventions should therefore adopt a dual focus: curbing excessive smartphone use while simultaneously enhancing self-control, time-management skills, and health awareness to sustain youths’ exercise involvement.

## 1 Introduction

### 1.1 The rise of mobile phone dependency and its behavioral implications

Smartphones have become nearly ubiquitous in adolescents’ lives, raising concerns about overuse and dependency. Previous studies suggests that approximately one in four adolescents exhibits symptoms of problematic smartphone use (Alimoradi et al., 2022; Sohn et al., 2019). Such mobile phone dependency is associated with a host of negative behavioral and health outcomes. Excessive smartphone use can disrupt healthy routines – for example, it often displaces time for physical activity, contributing to more sedentary behavior. Empirical studies have linked adolescent smartphone addiction to sleep disturbances, physical inactivity, poorer academic performance, and increased risks of overweight and musculoskeletal problems (Barr et al., 2018; Liu, 2023). Moreover, high phone use has been correlated with mental health issues such as depression, anxiety, and loneliness (Chen et al., 2021; Chen et al., 2022; Fung et al., 2021). Although some scholars debate whether “smartphone addiction” is a distinct clinical entity (Panova and Carbonell, 2018), there is broad agreement that overreliance on smartphones can impair adolescents’ self-regulation and well-being (Reiß et al., 2024; Vogel et al., 2021). In the context of behavioral health, these findings highlight the need to examine how mobile phone dependency might undermine healthy lifestyle behaviors – in particular, whether and how it affects adolescents’ engagement in exercise.

### 1.2 Adolescent exercise participation: a declining trend

Regular exercise during adolescence is widely recognized as vital for physical health, mental health, and developmental well-being. It improves fitness, cardiometabolic health, bone strength, and even cognitive and emotional outcomes in youth (World Health Organization, 2023). Beyond health benefits, participation in organized exercise or sports also fosters pro-social development and healthy habits – teens involved in sports tend to report more positive health behaviors and fewer risky behaviors (Ansari and Rizvi, 2023). Despite these advantages, adolescent exercise participation has been on the decline. Surveillance data indicate that the majority of adolescents worldwide do not meet recommended activity levels. For instance, a global study by the WHO found that 81% of 11–17-year-olds are insufficiently physically active (World Health Organization, 2023). In many countries, trends in dedicated exercise or sports participation have stagnated or worsened. In the United States, the prevalence of adolescents participating in school or club sports dropped from about 58.4% in 2011 to 57.4% in 2019 (Deng and Fan, 2022). This decline in structured exercise is concerning, as it comes at a time when youth physical inactivity is already at crisis levels (Guthold et al., 2020). It is important to clarify that the present study focuses specifically on structured exercise, defined as intentional, planned, and often organized forms of physical engagement such as sports, physical training sessions, or fitness programs. This stands in contrast to daily physical activity, which encompasses unstructured and incidental movements—such as walking between classes or performing household chores—that are less cognitively engaged and socially embedded. Structured exercise, by virtue of its goal-directed, time-bound, and socially regulated nature, bears greater theoretical relevance for adolescent development, particularly in the domains of behavioral self-regulation, routine maintenance, and identity formation. The observable global decline in structured exercise participation therefore raises not only public health concerns but also theoretical imperatives for understanding how contemporary digital life, including pervasive mobile phone dependency, may disrupt adolescents’ engagement in these developmentally consequential behaviors. The observable downward trend in adolescent exercise participation underscores an urgent need to understand factors that may be deterring youth from exercise in today’s media-saturated environment.

### 1.3 Theoretical account of behavioral mechanisms

To explore how mobile phone dependency might lead to reduced exercise participation, we draw on three complementary theoretical perspectives. Self-control theory posits that individuals possess a finite capacity for self-regulation, and that depleted self-control leads to preference for immediate rewards over long-term benefits (Freeman et al., 2013; Klinger et al., 2018). In the context of smartphone use, frequent phone distractions can deplete adolescents’ self-control resources, making it harder for them to stick with effortful behaviors like exercise. Indeed, research has found a reciprocal link between problematic smartphone use and self-control: adolescents who overuse phones tend to exhibit diminished self-discipline, and poor self-control in turn makes them more prone to addictive phone habits (Mao et al., 2022; Liu, 2023). This erosion of self-control may manifest as exercise avoidance, as youths impulsively choose phone entertainment over the more effortful choice of working out (Ye et al., 2025).

Next, time structure theory provides a framework for how distorted time management can undermine healthy routines. Time structure theory emphasizes that organized and meaningful allocation of time supports health-related behaviors, whereas unstructured time promotes procrastination and disorganization (Bond and Feather, 1988; Claessens et al., 2007). Smartphone overuse, however, often disrupts one’s sense of time and daily structure (Yuan et al., 2023). Adolescents absorbed in their phones may lose track of time or procrastinate on planned tasks (Chung et al., 2018). Studies show that smartphone addiction is linked to poor time management skills, with youth reporting difficulty in prioritizing tasks and adhering to schedules (Chen and Lyu, 2024; Chen et al., 2020; Liu et al., 2022; Zhao et al., 2025). According to this view, mobile phone dependency might weaken adolescents’ ability to implement exercise plans by fragmenting their time and reducing the regularity of their routines (Gong et al., 2023; Zhu et al., 2024). In essence, an adolescent may intend to exercise, but the lack of time structure – exacerbated by unstructured screen time – impairs the execution of those intentions.

Finally, we consider the lens of the Health Belief Model (HBM) to address motivational factors. The health belief model (HBM) suggests that engagement in health-promoting behaviors is influenced by perceived risks and benefits, self-efficacy, and cues to action (Houlden et al., 2021; Kim et al., 2022). If adolescents have a low health consciousness or weak health beliefs, they may not be motivated to be physically active. Smartphone-dependent teens might undervalue the long-term health risks of inactivity or the benefits of exercise, especially if immediate entertainment on their device is more salient. For example, an adolescent deeply engaged in mobile gaming or social media may give little consideration to the future health consequences of a sedentary lifestyle (Zhang et al., 2024). In HBM terms, the cues to action for exercise (like feeling out of shape or advice to be active) could be drowned out by digital distractions (Khodaveisi et al., 2021). Thus, diminished health awareness and priority – potentially a byproduct of high smartphone immersion – could be another pathway linking phone overuse to poor exercise participation. By integrating these three theoretical perspectives (self-control deficits, time disorganization, and low health motivation), we can form a more complete picture of the psychological mechanisms that might connect adolescent phone dependency to reduced exercise behavior.

### 1.4 Empirical gaps and conceptual limitations

Although prior studies have begun examining links between smartphone use and physical activity, several important gaps remain. First, most existing research has been variable-centric, relying on linear correlations or regressions between individual variables. This variable-oriented approach tends to overlook the interactive mechanisms and complex configurations that might be at play. In reality, phone overuse, self-control, time management, and health motivation likely influence exercise behavior in combination. Traditional analyses may not capture these interdependencies. Second, the mediating factors underlying the smartphone–exercise link have not been explored in a systematic or comprehensive way. Different studies have identified isolated mediators – for example, stress, loneliness, or social support – in fragmentary pathways, but the field lacks an integrated understanding of how multiple mediators work together. There is evidence that the direct association between smartphone use and physical activity is weak or inconsistent, implying that intervening variables are crucial. For instance, some studies have identified isolated mediators, such as social anxiety (Song et al., 2024), resilience (Wu et al., 2024), or psychological climate (Dong et al., 2025), yet these findings remain disconnected. The result is a patchwork of possible mechanisms without a unifying model. Third, and relatedly, there is a lack of a unified theoretical framework that brings together the multiple psychological mechanisms in the “smartphone dependency → exercise behavior” relationship. To date, no single study has simultaneously considered self-control, time structure, and health beliefs (or similar constructs) in explaining why adolescents who are glued to their phones might skip exercise. The absence of an integrative model means that we do not fully understand the joint influence of these factors, nor which factors might be most pivotal. In sum, prior research has typically examined pieces of the puzzle in isolation, leaving conceptual and empirical blind spots regarding how smartphone dependence impacts adolescent exercise engagement.

### 1.5 The present study

**Figure 1:**
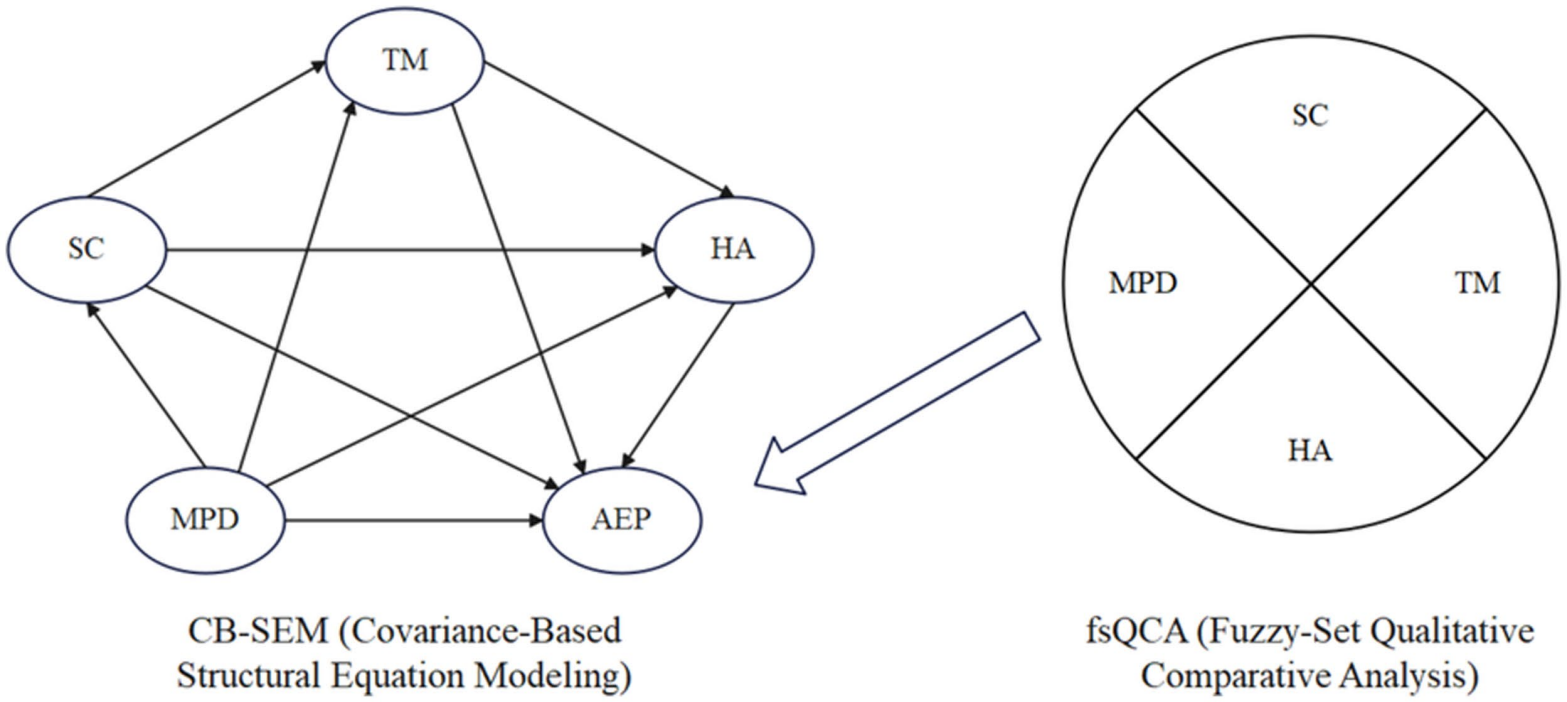
Conceptual model of this study.

The present study aims to address these gaps by proposing and testing a comprehensive model that links mobile phone dependency to adolescents’ exercise participation through multiple psychological pathways. Figure 1 illustrates the conceptual framework. We hypothesize that high mobile phone dependency will be associated with lower exercise participation, and that this relationship is mediated through three key mechanisms: (a) diminished self-control capacity, (b) disrupted time management (poor time structure), and (c) reduced health motivation (weak exercise-related health beliefs). In our model, each of these mediators represents a distinct explanatory route grounded in the theories discussed above. By integrating them, we seek to provide a more holistic account of the behavioral mechanism from smartphone overuse to exercise avoidance.

To test this model, we employ a structural equation modeling (SEM) approach. SEM allows us to examine the multiple mediating pathways simultaneously and to evaluate the overall fit of the proposed model to the data. This analytic strategy goes beyond simple pairwise correlations, enabling us to ascertain the unique contribution of each mediator while controlling for the others. We will be able to determine, for example, whether self-control deficits and time disorganization each explain unique variance in exercise behavior, or whether one pathway dominates. In addition, recognizing that adolescents’ behavior might result from combinations of factors (rather than single factors in isolation), we complement the variable-centered SEM with a fuzzy-set qualitative comparative analysis (fsQCA). The fsQCA technique will enable us to identify specific configurations of conditions that are sufficient for low exercise participation outcomes. For instance, we can explore if the convergence of high phone dependency and low self-control and low health motivation produces a “critical combination” that consistently yields poor exercise rates, as opposed to other adolescents who might be high in phone use but remain active due to protective factors. This configurational analysis provides a person-centered perspective to complement the SEM’s variable-centered insights.

In summary, the present study has two primary objectives: (1) to quantitatively test a theory-driven model in which mobile phone dependency influences adolescent exercise participation through multiple psychological mediators, and (2) to qualitatively examine the combinations of factors that lead to diminished exercise behavior using a configurational approach. By doing so, we endeavor to contribute a more nuanced understanding of how smartphone dependency can erode youths’ exercise involvement, integrating self-regulatory, temporal, and motivational dimensions. Ultimately, our goal is to inform intervention efforts by pinpointing not only individual influential pathways but also the profiles of adolescents who may be most at risk of physical inactivity due to their pattern of smartphone use and psychological characteristics.

## 2 Method

### 2.1 Participants and procedure

The data were collected through a cross-sectional survey administered to adolescents in Grades 5 through 9 across various schools in China. A total of 1,500 questionnaires were distributed using stratified cluster sampling, targeting both rural and urban school settings. After excluding incomplete or invalid responses (e.g., straight-lining, missing responses), 1,404 valid questionnaires remained, yielding a valid response rate of 93.60%.

To evaluate the adequacy of the sample size, two commonly used criteria were applied: the item-based ratio and the variable-based ratio. The survey instrument contained a total of 101 items across 19 dimensions, encompassing constructs such as mobile phone dependency (MPD), self-control (SC), time management (TM), health awareness (HA), and adolescent exercise participation (AEP). Following the recommended 5:1 to 10:1 participant-to-item ratio (Hatcher, 1994; Suhr, 2006), the minimum required sample size would range from 505 to 1,010. Additionally, applying the 10:1 to 20:1 participant-to-variable ratio for structural equation modeling (Hair et al., 2013; Kline, 2016), a sample size of 190 to 380 would suffice for the 19 observed variables. The final valid sample of 1,404 participants substantially exceeded both thresholds, ensuring adequate statistical power and reliable parameter estimation. Demographic details of the participants are presented in Table 1.

**Table 1:** Demographic characteristics of the sample (*n* = 1,404).

Basic information	Category	Frequency	Percentage
Gender	Male	699	49.79
	Female	705	50.21
Grade	Grade 5	288	20.51
	Grade 6	361	25.71
	Grade 7	371	26.42
	Grade 8	265	18.87
	Grade 9	119	8.48
School location	Rural	694	49.43
	Urban	710	50.57

### 2.2 Measures

Mobile phone dependency (MPD) was assessed using a 17-item scale developed by Leung (2008), consisting of four dimensions: loss of control, withdrawal, escape, and inefficiency. Adolescent exercise participation (AEP) was measured with a 3-item scale adapted from Liang (1994), covering exercise intensity, frequency, and duration. Self-control (SC) was measured using a 19-item instrument developed by Tan and Guo (2008), which includes five dimensions: impulse control, healthy habits, entertainment restraint, work focus, and resistance to temptation. Time management (TM) was assessed using the 44-item Time Management Disposition Scale by Huang and Zhang (2001), comprising three dimensions: value of time, time monitoring, and time efficacy. Health awareness (HA) was evaluated using an 18-item scale based on the Health Belief Model, developed by Sun et al. (2024), encompassing health consciousness, perceived susceptibility, perceived severity, and self-efficacy.

All constructs were measured using previously validated instruments that have been adapted for use with Chinese adolescents. Each scale employed a 5-point Likert response format, ranging from 1 (strongly disagree) to 5 (strongly agree), with higher scores indicating greater levels of the corresponding construct.

### 2.3 Analytical strategy

#### 2.3.1 Structural equation modeling

Covariance-based structural equation modeling (CB-SEM) was conducted using SPSS 27.0 and AMOS 26.0 to examine the hypothesized relationships among mobile phone dependency (MPD), self-control (SC), time management (TM), health awareness (HA), and adolescent exercise participation (AEP). The analytic procedure included descriptive statistics, reliability and validity testing, common method bias assessment, model estimation, mediation analysis, and robustness checks.

Descriptive statistics, Pearson correlations, and internal consistency coefficients were computed in SPSS 27.0. Cronbach’s alpha values ≥ 0.70 were considered acceptable indicators of reliability (Tavakol and Dennick, 2011). Confirmatory factor analysis (CFA) was performed in AMOS 26.0 to examine construct validity. Convergent validity was assessed using standardized factor loadings (≥0.60), average variance extracted (AVE ≥ 0.50), and composite reliability (CR ≥ 0.70), while discriminant validity was evaluated by comparing the square root of AVE with inter-construct correlations (Fornell and Larcker, 1981). Common method bias was examined through Harman’s single-factor test and confirmatory model comparisons (Podsakoff et al., 2003). Structural model fit was evaluated based on established thresholds: *χ*^2^/df ≤ 5.00, CFI ≥ 0.90, TLI ≥ 0.90, RMSEA ≤ 0.08, and SRMR ≤ 0.08 (Kline, 2016). Mediation effects were tested using bias-corrected bootstrapping with 2,000 resamples and 95% confidence intervals (Kline, 2016).

Robustness checks were conducted using hierarchical multiple regression analyses in SPSS 27.0. Control variables were entered in the first step, followed by theoretical predictors in the second step, to assess the consistency of the SEM findings under an alternative estimation framework. Multicollinearity was examined using variance inflation factor (VIF), with values below 5.0 indicating acceptable levels (O’Brien, 2007).

#### 2.3.2 Fuzzy-set qualitative comparative analysis

Fuzzy-set qualitative comparative analysis (fsQCA) was employed using fsQCA 3.0 to explore multiple causal configurations leading to high levels of adolescent exercise participation. The procedure consisted of three main steps: data calibration, necessity condition analysis, and truth table construction. All continuous variables were transformed into fuzzy sets using the direct calibration method (Ragin, 2009), with three qualitative anchors: full membership (0.95), crossover point (0.50), and full non-membership (0.05). Necessity condition analysis was conducted with a consistency threshold of 0.90. Subsequently, a truth table was generated, and sufficiency analysis was performed using a consistency threshold of 0.80, a PRI threshold of 0.70, and a minimum frequency threshold of 1 case (Fiss, 2011).

To ensure the robustness of the fsQCA findings, three sensitivity tests were performed (Schneider and Wagemann, 2012; Judge et al., 2020): (1) calibration anchors were relaxed from 0.95/0.50/0.05 to 0.75/0.50/0.25; (2) the case frequency threshold was increased from 1 to 2 and further to 22 (approximately 1.5% of the total sample); and (3) the consistency threshold for identifying sufficient configurations was raised from 0.80 to 0.85. The stability of core configurations across these settings was examined to verify the reliability of the configurational solutions.

## 3 Results

### 3.1 Preliminary analyses

#### 3.1.1 Descriptive statistics, reliability, validity, and correlational analysis

As shown in Table 2, all five variables demonstrated acceptable psychometric properties. The Cronbach’s *α* coefficients ranged from 0.902 (AEP) to 0.958 (SC), and all subscales also met acceptable internal consistency thresholds. Standardized factor loadings for each construct exceeded 0.60, and the composite reliability (CR) values ranged from 0.756 to 0.876, all above the recommended 0.70 cutoff. Average variance extracted (AVE) ranged from 0.512 to 0.639, confirming convergent validity.

**Table 2:** Descriptive statistics, internal consistency reliability, and convergent validity of study variables.

Variable	*M*	SD	Cronbach’s α (range)	Std. factor loading	CR	AVE
MPD	2.44	0.76	0.940 (0.808–0.924)	0.703–0.820	0.845	0.578
AEP	3.54	0.79	0.902	0.645–0.839	0.756	0.512
SC	3.58	0.69	0.958 (0.897–0.920)	0.741–0.769	0.873	0.578
TM	3.56	0.85	0.925 (0.895–0.923)	0.784–0.812	0.778	0.637
HA	3.54	0.78	0.916 (0.801–0.875)	0.781–0.810	0.876	0.639

M, mean; SD, standard deviation; Cronbach’s α (Range) reports the internal-consistency coefficient for the full scale, followed in parentheses by the minimum and maximum Cronbach’s α values across its first-order subscales; Std. factor loading = standardized factor-loading range obtained from the confirmatory factor analysis.

Table 3 reports the bivariate correlations and the results of discriminant validity testing. All correlations were significant at *p* < 0.001. The square root of AVE for each construct (diagonal entries) exceeded its correlations with other constructs, satisfying the Fornell–Larcker criterion for discriminant validity.

**Table 3:** Correlations and assessment of discriminant validity.

Variable	MPD	AEP	SC	TM	HA
MPD	**0.760**				
AEP	−0.478^***^	**0.716**			
SC	−0.460^***^	0.554^***^	**0.760**		
TM	−0.480^***^	0.583^***^	0.575^***^	**0.798**	
HA	−0.476^***^	0.661^***^	0.522^***^	0.577^***^	**0.799**

Values on the diagonal (in bold) indicate the square roots of average variance extracted (AVE) for each construct. A discriminant validity criterion is met when the square root of AVE (diagonal value) exceeds the corresponding off-diagonal correlations. ^***^p < 0.001.

#### 3.1.2 Common method bias assessment

To assess common method bias (CMB), both exploratory and confirmatory approaches were applied. Harman’s single-factor test extracted 18 factors with eigenvalues above 1, with the first factor accounting for 28.260% of the variance—well below the 40% threshold—suggesting no substantial CMB.

As shown in Table 4, confirmatory factor analyses further compared measurement models. The one-factor model showed poor fit (*χ*^2^ = 3786.177, df = 152, CFI = 0.689, TLI = 0.650, SRMR = 0.136, RMSEA = 0.131), whereas the five-factor model corresponding to theoretical constructs showed good fit (*χ*^2^ = 386.627, df = 142, CFI = 0.979, TLI = 0.975, SRMR = 0.036, RMSEA = 0.035). The six-factor model with an additional method factor improved fit slightly (*χ*^2^ = 319.380, df = 141, CFI = 0.982, TLI = 0.980, SRMR = 0.032, RMSEA = 0.031), but the gain was minimal. Chi-square difference tests confirmed that the one-factor model was significantly inferior (*p* < 0.05). Overall, the results indicate that common method bias is not a serious concern in this study.

**Table 4:** Model fit indices from confirmatory factor analyses for common method bias assessment.

Model	*χ* ^2^	df	*χ*^2^/df	CFI	TLI	SRMR	RMSEA (90%CI)
One-factor model	3786.177	152	24.909	0.689	0.650	0.136	0.131 (0.127–0.134)
Five-factor model	386.627	142	2.723	0.979	0.975	0.036	0.035 (0.031–0.039)
Six-factor model	319.380	141	2.265	0.982	0.980	0.032	0.031 (0.024–0.033)

The one-factor model specifies all items loading onto a single latent factor, representing a common method. The five-factor model specifies each set of items loading onto their respective theoretical constructs. The six-factor model adds a latent method factor in addition to the six theoretical factors.

### 3.2 Structural equation modeling results

#### 3.2.1 Model fit and path analysis

**Figure 2:**
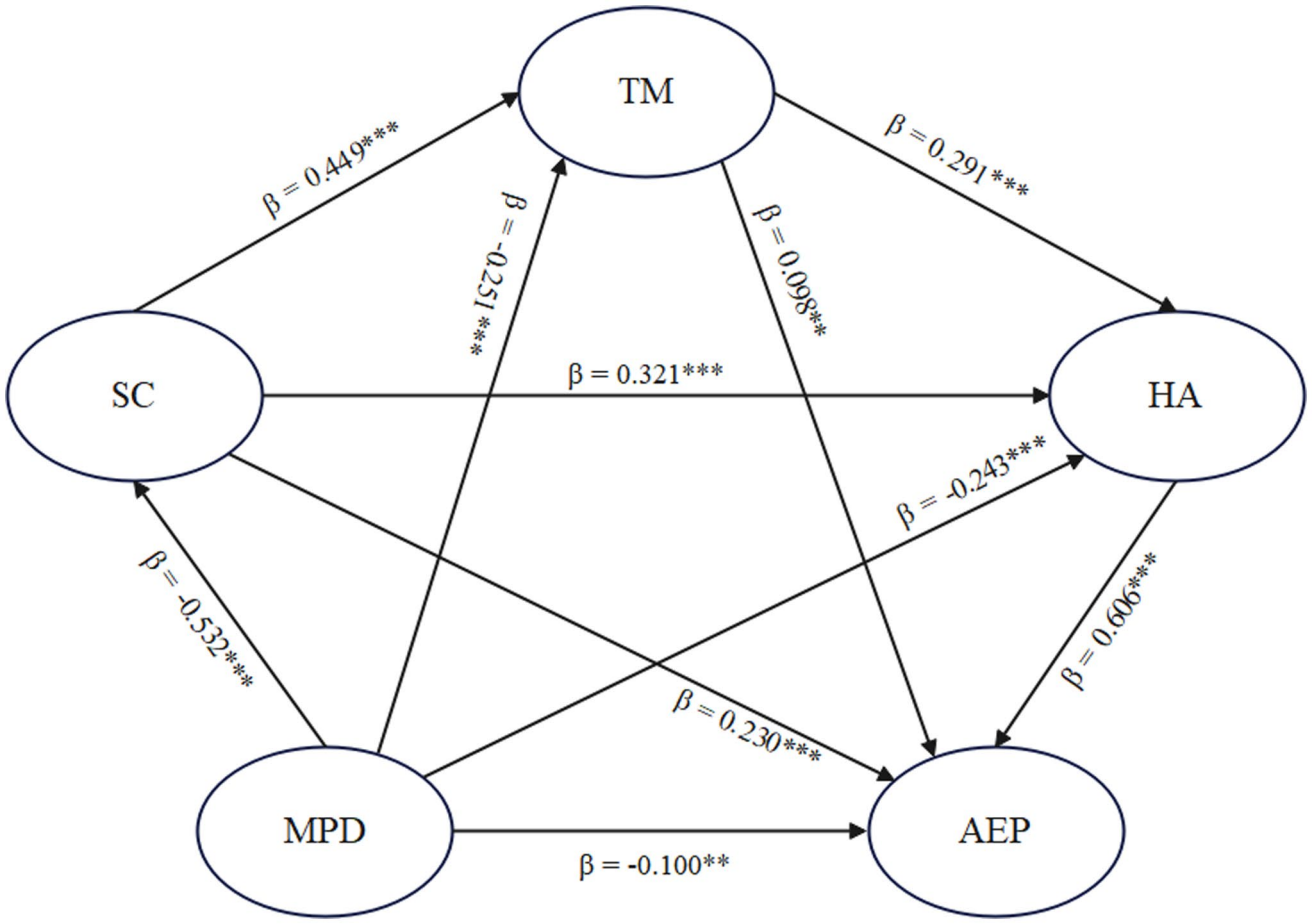
Standardized path coefficients of the structural equation model. ^**^p < 0.01, ^***^p < 0.001.

The hypothesized structural equation model was tested using AMOS 26.0, and the results indicated a good fit to the data: *χ*^2^/df = 2.723, CFI = 0.979, TLI = 0.975, SRMR = 0.036, RMSEA = 0.035, 90% CI [0.031, 0.039]. These fit indices meet the recommended cutoffs, supporting the structural validity of the proposed model. Figure 2 presents the standardized path coefficients among mobile phone dependency, self-control, time management, health awareness, and adolescent exercise participation. All hypothesized paths were estimated simultaneously, and all path coefficients reached statistical significance at the *p* < 0.05 level.

#### 3.2.2 Mediation analysis

To examine the mediating roles of self-control (SC), time management (TM), and health awareness (HA), a multiple mediation model was tested using the bias-corrected bootstrap method with 2,000 resamples. The results are presented in Table 5.

**Table 5:** Total, direct and indirect effects in the multiple mediator model.

Path	*β*	Boot SE	Boot LLCI	Boot ULCI	Ratio
Direct effect					
MPD → AEP	−0.100^**^	0.036	−0.172	−0.031	16.50%
Indirect effects					
MPD → SC → AEP	−0.122^**^	0.021	−0.162	−0.083	20.13%
MPD → TM → AEP	−0.025^**^	0.010	−0.047	−0.007	4.13%
MPD → HA → AEP	−0.147^**^	0.024	−0.195	−0.101	24.26%
MPD → SC → TM → AEP	−0.023^**^	0.009	−0.043	−0.007	3.80%
MPD → SC → HA → AEP	−0.103^**^	0.014	−0.133	−0.078	16.99%
MPD → TM → HA → AEP	−0.044^**^	0.009	−0.064	−0.030	7.26%
MPD → SC → TM → HA → AEP	−0.042^**^	0.007	−0.057	−0.030	6.93%
Total indirect effects					83.50%
Total effect	−0.606^***^	0.029	−0.661	−0.547	100%

β = standardized path coefficient; Boot SE = bootstrap standard error; Boot LLCI = the lower bound of the 95% confidence interval; Boot ULCI = the upper limit of the 95% confidence interval (Percentile Bootstrap Method with Bias Correction). The Bootstrap sample size is set at 2000. ^**^p < 0.01, ^***^p < 0.001.

The total effect of mobile phone dependency (MPD) on adolescent exercise participation (AEP) was significant (*β* = −0.606, *p* < 0.001), with a 95% confidence interval that did not include zero [−0.661, −0.547]. The direct effect remained significant (*β* = −0.100, *p* < 0.01), accounting for 16.50% of the total effect, indicating partial mediation.

A total of seven indirect paths were identified, all of which reached significance at the 0.01 level. Among the single-step mediation paths, the effect through HA was the strongest (*β* = −0.147), followed by SC (*β* = −0.122) and TM (*β* = −0.025). In addition, three two-step chained mediation paths were significant: MPD → SC → TM → AEP (*β* = −0.023), MPD → SC → HA → AEP (*β* = −0.103), and MPD → TM → HA → AEP (*β* = −0.044). A three-step sequential path—MPD → SC → TM → HA → AEP—also showed a significant indirect effect (*β* = −0.042). The combined indirect effects accounted for 83.50% of the total effect. These results confirm the presence of multiple and chained mediation mechanisms linking mobile phone dependency to exercise behavior among adolescents.

#### 3.2.3 Robustness test

To assess the robustness of the structural model findings, a hierarchical multiple regression analysis was conducted using SPSS 27.0. Two models were estimated with adolescent exercise participation (AEP) as the dependent variable. Model 1 included only the control variables (gender, grade, and school location), while Model 2 introduced the core predictors—mobile phone dependency (MPD), self-control (SC), time management (TM), and health awareness (HA). The results are presented in Table 6.

**Table 6:** Robustness test of core predictors on adolescent exercise participation: hierarchical regression results.

Variable	Dependent variable (AEP)	
	Model 1	Model 2
Gender	0.001	−0.017
Grade	0.083^**^	0.047^*^
School location	−0.006	0.002
MPD		−0.092^***^
SC		0.180^***^
TM		0.190^***^
HA		0.418^***^
*R* ^2^	0.007	0.535
Adjusted *R*^2^	0.005	0.532
*F*	3.241^*^	229.113^***^

Model 1 includes only control variables; Model 2 adds all key predictors. ^*^p < 0.05, ^**^p < 0.01, ^***^p < 0.001.

In Model 1, grade was a significant predictor of AEP (*β* = 0.083, *p* < 0.01), while gender and school location were not significant. The model explained a negligible portion of variance (*R*^2^ = 0.007). In Model 2, after adding the theoretical variables, the explained variance increased substantially (Δ*R*^2^ = 0.528), with the full model accounting for 53.5% of the variance in AEP (Adjusted *R*^2^ = 0.532, *F* = 229.113, *p* < 0.001). All key predictors showed significant effects in the expected directions: MPD negatively predicted AEP (*β* = −0.092, *p* < 0.001), whereas SC (*β* = 0.180, *p* < 0.001), TM (*β* = 0.190, *p* < 0.001), and HA (*β* = 0.418, *p* < 0.001) were positively associated with AEP. These findings confirm the robustness of the structural relationships identified in the SEM analysis, demonstrating their stability under an alternative estimation approach.

### 3.3 Fuzzy-set qualitative comparative analysis results

#### 3.3.1 Necessity condition analysis

A necessity analysis was conducted to examine whether any single condition consistently leads to high or non-high adolescent exercise participation (AEP). As shown in Table 7, none of the conditions—nor their negations—reached the conventional consistency threshold of 0.90, indicating that no individual factor constitutes a necessary condition for either outcome. This suggests that adolescent exercise participation is driven by configurations of multiple conditions rather than any single factor.

**Table 7:** Necessary condition analysis for high and non-high adolescent exercise participation (AEP).

Condition	High AEP		Non-high AEP	
	Consistency	Coverage	Consistency	Coverage
MPD	0.511	0.549	0.745	0.726
~MPD	0.745	0.763	0.537	0.500
SC	0.751	0.781	0.517	0.488
~SC	0.508	0.537	0.768	0.737
TM	0.773	0.782	0.522	0.479
~TM	0.485	0.528	0.762	0.753
HA	0.775	0.817	0.487	0.466
~HA	0.493	0.514	0.808	0.766

“~” denotes the absence or low level of a given condition.

#### 3.3.2 Conditional configuration analysis

The configuration analysis identified three sufficient configurations for high adolescent exercise participation (AEP) and one for non-high AEP, as summarized in Table 8.

**Table 8:** Configurational solutions for achieving high and non-high adolescent exercise participation (AEP).

Conditions	High AEP			Non-high AEP
	S1	S2	S3	NS1
MPD	⨂	⨂		●
SC	●		●	⨂
TM		●	●	⨂
HA	●	●	●	⨂
Consistency	0.914	0.917	0.915	0.918
Raw coverage	0.544	0.561	0.567	0.513
Unique coverage	0.043	0.060	0.066	0.513
Solution consistency	0.887		0.513	
Solution coverage	0.670		0.918	

● indicates the presence of a core condition; ⨂ indicates the absence of a core condition; blank cells indicate that the condition may be either present or absent.

For high AEP, low mobile phone dependency (MPD) emerged as a critical factor in two configurations. In S1, the absence of MPD combined with high self-control (SC) and health awareness (HA) to form a sufficient path. In S2, the absence of MPD paired with strong time management (TM) and HA also led to high AEP. These two solutions reflect that low MPD functions as a core facilitating condition, especially when coupled with specific psychological resources.

In contrast, S3 represents a distinct pathway in which MPD plays no determining role—it appears as a “do not care” condition—suggesting that when SC, TM, and HA are all strongly present, high AEP can be achieved regardless of MPD level. This highlights the possibility that robust internal and cognitive strengths can compensate for mobile phone use, making its presence or absence less critical in certain configurations.

For non-high AEP, one configuration (NS1) was identified. This solution involves the presence of high MPD, coupled with the absence of SC, TM, and HA. With a consistency of 0.918 and unique coverage of 0.513, NS1 illustrates a typical risk-oriented pathway, where excessive mobile phone use in combination with a lack of self-regulatory and health resources significantly undermines physical activity.

Overall, these results underscore the configurational asymmetry between promoting and inhibiting conditions of adolescent exercise participation. While low MPD is a recurrent component in effective configurations, high AEP may still occur in its presence if sufficient internal resources exist. Conversely, high MPD becomes problematic only when not buffered by compensatory mechanisms.

#### 3.3.3 Robustness test

To examine the stability of the configurational results, three robustness tests were conducted by systematically adjusting key analytical parameters. First, the calibration anchors were relaxed from the original thresholds (0.95, 0.50, 0.05) to a more lenient set (0.75, 0.50, 0.25). Second, the minimum frequency threshold was increased from 1 to 2 and further to 22 (approximately 1.5% of the total sample). Third, the consistency threshold for identifying sufficient configurations was raised from 0.80 to 0.85. Across all three sensitivity checks, the original configurations for both high and non-high adolescent exercise participation (AEP) remained unchanged. These results demonstrate that the identified configurational solutions are robust and not sensitive to variations in calibration or model parameters, thereby enhancing the credibility of the fsQCA findings.

## 4 Discussion

### 4.1 Structural equation modeling results: direct and mediated associations with MPD

The structural equation modeling results supported our hypotheses regarding the negative influence of mobile phone dependency (MPD) on adolescents’ exercise participation (AEP). Specifically, MPD was found to be a significant negative predictor of AEP, indicating that adolescents who are more dependent on their mobile phones tend to engage in less physical exercise. This result aligns with prior studies reporting that excessive smartphone use is associated with reduced physical activity and poorer health indicators in youth (Barr et al., 2018; Chen et al., 2021; Chen et al., 2022; Fung et al., 2021; Kim et al., 2015; Liu, 2023). A recent meta-analysis further corroborated this association, reporting a moderate overall negative correlation (around *r* = −0.24) between mobile phone addiction and physical activity among young people (Xiao et al., 2022), which is consistent with the sizable total effect observed in our model (*β* = −0.606). Notably, both the direct effect of MPD on AEP (*β* = −0.100, *p* < 0.01) and the total indirect effects through psychological mediators were statistically significant. These findings indicate that mobile phone dependency undermines adolescents’ exercise behavior through a combination of direct behavioral mechanisms and indirect psychological processes, including diminished self-control, time disorganization, and weakened health awareness.

Among the three mediators, health awareness (HA) emerged as the most prominent pathway (indirect *β* ≈ −0.15), suggesting that motivational disengagement plays a central role in how smartphone overuse undermines exercise behavior. Adolescents with high levels of MPD may develop a lower sense of urgency about maintaining physical health, reduced sensitivity to long-term risks of inactivity, or diminished prioritization of exercise as a lifestyle choice. These tendencies are likely shaped by sustained attention toward screen-based stimuli at the expense of internalized health goals. This interpretation is consistent with findings that adolescents who lack health consciousness or basic health knowledge are less likely to engage in physical activity, and that digital distraction may suppress both the salience and accessibility of health-related intentions (Khodaveisi et al., 2021; Zhang et al., 2024). When health beliefs are weakened, behavioral activation toward exercise becomes less likely.

The second strongest indirect effect was observed through self-control (SC) (*β* ≈ −0.12), indicating that regulatory competence is a key personal resource protecting against the behavioral costs of digital overexposure. Adolescents with diminished self-control are more vulnerable to immediate digital gratifications and less capable of executing sustained, effortful behaviors such as physical activity. This finding aligns with research showing that higher self-control is associated with effective resistance to smartphone temptation and greater behavioral consistency (Berger et al., 2018). Prior studies have also demonstrated that adolescents with better regulatory capacity are more likely to adopt coping strategies to limit problematic phone use, while those with low self-control display higher impulsivity and are more susceptible to adverse behavioral outcomes, including physical inactivity (Dontre, 2021; Hartley et al., 2022). The SC pathway thus reflects a disruption in executive functioning, whereby poor behavioral regulation compromises adherence to exercise routines.

Although time management (TM) showed the smallest single indirect effect (*β* ≈ −0.03), its role remains theoretically important as a proximal enabler of behavioral execution. Excessive phone use may fragment adolescents’ daily structure, displace planned activity windows, or interfere with the temporal coordination necessary for consistent exercise. Rather than representing a lack of intention, poor time management reflects failures in planning, prioritization, and schedule adherence. This interpretation is supported by findings that adolescents with higher smartphone dependency often report difficulty in organizing daily tasks, adhering to structured routines, and allocating time effectively (Chung et al., 2018; Liu et al., 2022; Yuan et al., 2023). While the effect size was relatively modest, the TM pathway highlights the practical consequences of digital distraction in diminishing one’s capacity to implement health-related intentions in time-bound environments.

In addition to these single mediation effects, the model identified three significant two-step chained pathways and one three-step sequential pathway, together accounting for approximately 22% of the total effect. These results reflect the interdependent nature of the mediators and suggest that MPD undermines adolescent exercise not only through isolated mechanisms, but also through dynamic sequences of psychological erosion. The indirect path MPD → SC → HA → AEP (*β* = −0.103) indicates that a loss of regulatory control may precede and exacerbate motivational disengagement, thereby forming a dual-risk pathway. Similarly, the chain MPD → SC → TM → AEP (*β* = −0.023) suggests that failures in behavioral regulation may give rise to temporal disorganization, which further reduces physical activity engagement. The path MPD → TM → HA → AEP (*β* = −0.044) illustrates how chaotic time use may impair health awareness and devalue the perceived urgency or importance of exercise. The full sequential chain MPD → SC → TM → HA → AEP (*β* = −0.042) captures a cascading process in which impaired self-control leads to disorganized time use, which in turn weakens health motivation, ultimately resulting in reduced physical activity. These chained paths provide a nuanced picture of how digital dependency may trigger progressive degradation across multiple behavioral systems, offering a more ecologically valid account of adolescent behavioral decline in media-saturated environments.

### 4.2 Configurational results: multiple pathways to high and non-high AEP

Beyond the variable-centered insights above, our fuzzy-set qualitative comparative analysis (fsQCA) revealed multiple combinatorial pathways leading to the outcome of high adolescent exercise participation, as well as a distinct pathway leading to low participation. This configurational approach offers a complementary perspective, highlighting the principle of equifinality (Douglas et al., 2020; Fiss, 2011) – that is, there are multiple different sets of conditions that can produce an equivalent outcome (in this case, high physical activity). Consistent with this notion, the necessity analysis showed that no single factor (neither the presence nor absence of MPD, SC, TM, or HA) was indispensable for achieving high exercise levels – all consistency scores were well below the 0.90 threshold. In other words, there is no “silver bullet” condition that guarantees an active lifestyle; instead, exercise behavior arises from configurations of several factors acting in concert. This validates the multifactorial view presented in our introduction and is in line with arguments in the literature that focusing only on isolated variables can overlook important interactions and contextual factors in health behaviors (Fan et al., 2025). Our QCA results explicitly capture these interactions by identifying which combinations of conditions are sufficient to foster high AEP or, conversely, to result in low AEP.

For high exercise participation, we identified three distinct sufficient configurations (S1–S3), each representing a different recipe for success. Notably, two solutions (S1 and S2) featured the absence of mobile phone dependency (⨂ MPD) as a core condition, combined with strong personal resources. In S1, adolescents who were low in MPD and exhibited high self-control and high health awareness were very likely to have high exercise involvement. In S2, low MPD paired with high time management and high health awareness also led to the outcome. These findings underscore that keeping smartphone habits under control can strongly facilitate physical activity – especially when youths also possess the self-regulatory skills or time-organizing abilities to take advantage of their low-distraction environment. Stated differently, minimizing mobile phone overuse appears to create a fertile context in which positive attributes like self-discipline, scheduling/planning skills, and health consciousness can translate into actual exercise behavior. This agrees with our earlier mediation finding that high MPD hinders exercise largely by eroding those same personal attributes. Here, QCA shows the flip side: low MPD can enable those attributes to positively influence behavior, as reflected by S1 and S2 pathways. However, an even more interesting insight emerges from the third solution, S3, which did not include MPD as a determining condition. In S3, adolescents attained high physical activity despite any level of phone use, so long as they simultaneously had high self-control, high time management, and high health awareness. This configuration suggests a scenario where strong internal resources can compensate for, or override, the potential negative influence of mobile phone dependency. An adolescent with exceptional self-control, good time management habits, and a keen awareness of health may successfully maintain frequent exercise even if they happen to use smartphones heavily – they might, for instance, strictly schedule their workouts and resist phone distractions during those times, driven by their health priorities. In essence, a surplus of self-regulatory strength and health motivation can neutralize the downsides of MPD. This finding resonates with other research indicating that personal strengths (like self-control or resilience) can buffer the adverse effects of high screen time (Berger et al., 2018). It also echoes the idea of equifinality: there is more than one path to being active – some adolescents achieve it by avoiding risk factors (low MPD) in combination with a couple of supporting factors, whereas others achieve it by actively counterbalancing a risk factor (high MPD) with an even stronger presence of multiple protective factors (high SC, TM, HA). Importantly, all three high-AEP configurations shared one common ingredient: high health awareness. This consistency highlights health consciousness as a particularly crucial driver of adolescent exercise. When young people recognize the importance of physical activity for their well-being, they are much more likely to prioritize and sustain exercise – even to the point of mitigating other hindrances. This aligns with recent findings that health awareness significantly predicts exercise behavior in youth (Liang et al., 2023). In practical terms, it suggests that interventions aiming to raise adolescents’ health awareness (e.g., through health education campaigns or fitness goal setting) could be a universally beneficial strategy, regardless of other circumstances.

In contrast to the multiple pathways for high exercise, the fsQCA identified only one predominant configuration leading to non-high exercise participation, labeled NS1. This configuration can be characterized as the “risk-loaded” pathway: it involves the presence of high MPD along with the absence of self-control, time management, and health awareness (● MPD, ⨂ SC, ⨂ TM, ⨂ HA). In our sample, more than half of the low-active adolescents matched this profile (raw coverage 0.513), indicating that this combination of excessive phone use and poor self-regulatory and health orientations is a common recipe for disengagement from physical activity. The consistency of NS1 was very high (0.918), meaning that adolescents exhibiting this risk profile were almost always in the low exercise group. This finding reinforces the concern that heavy mobile phone dependency can be truly detrimental when not buffered by any protective factors. In such cases, smartphone overuse likely consumes time and attention while also coinciding with a lack of personal discipline or concern for health, thereby dramatically reducing the likelihood of engaging in sports or exercise. We see here a clear asymmetry in the conditions for high vs. low exercise: while low MPD is not absolutely required for an active lifestyle (as shown by S3), high MPD combined with low SC, TM, and HA is a very potent sufficient condition for an inactive lifestyle. In other words, phone addiction by itself does not doom an adolescent to inactivity in every case – but if it is accompanied by deficient self-regulation and health values, inactivity becomes highly likely. This asymmetrical pattern is consistent with the idea that protective factors can outweigh risk factors in positive outcomes, but in their absence, the risk factors’ full negative impact manifests. It also underscores that the determinants of physical inactivity are not simply the mirror opposite of those for activity (since multiple active paths exist, but the dominant inactive path is essentially the converse of the most resource-rich active path).

Overall, the configurational results deepen our understanding of how different factors intersect to influence adolescent exercise behavior. They demonstrate that promoting youth physical activity may require jointly addressing several influences rather than focusing on any single factor in isolation. This methodological triangulation with QCA complements the SEM findings: the regression-based SEM confirmed average linear relationships (e.g., MPD generally undermines exercise, and SC/TM/HA facilitate it), whereas the fsQCA reveals the nuanced combinations underlying those general trends. Notably, our sensitivity checks showed that these configurations were robust to various analytical adjustments (e.g., changing calibration thresholds and consistency cutoffs), lending credibility to the patterns identified. By using both approaches, we capture a fuller picture of the phenomenon – the mediational analysis quantified the proportion of MPD’s effect explained by certain mediators, and the configurational analysis uncovered alternative pathways and context-dependent effects. This approach addresses the complexity in adolescent behavior that traditional one-variable-at-a-time models might miss (Fan et al., 2025). It reinforces that intervention strategies should be multidimensional and tailored, a point we turn to next.

## 5 Practical implications

### 5.1 Interventions for adolescents: enhancing internal capacities

At the individual level, the findings highlight the importance of strengthening adolescents’ internal self-regulatory and motivational resources. The SEM results point to self-control (SC), time management (TM), and health awareness (HA) as critical mediators between mobile phone dependency (MPD) and exercise behavior. Therefore, intervention efforts should focus on bolstering these capacities directly. For SC, programs may incorporate self-monitoring routines, impulse delay techniques, or mindfulness-based training to improve adolescents’ capacity to resist digital temptations and persist with exercise plans. For TM, schools or youth organizations could offer planning workshops or behavioral time-blocking methods to help adolescents allocate time more deliberately for physical activity amidst competing screen demands. To elevate HA, health education should extend beyond generic messaging, emphasizing personal relevance and long-term consequences of inactivity. These strategies correspond to self-control theory, time structure theory, and the health belief model, respectively. Furthermore, fsQCA revealed that certain adolescents (e.g., Profile S3) maintained high activity levels despite MPD by mobilizing compensatory internal strengths; such resilient individuals could be identified and engaged as peer mentors or role models in activity promotion programs.

### 5.2 Family-level strategies: structuring the home environment

Parents and guardians play a pivotal role in shaping adolescents’ digital habits and health routines. Interventions at the family level should focus on environmental scaffolding and co-regulatory practices. Consistent with time structure theory, families might introduce structured daily routines that incorporate fixed times for physical activity and limit unstructured screen exposure. Instead of solely imposing screen-time bans, parents could adopt collaborative strategies such as establishing shared physical goals (e.g., evening walks, weekend sports) and applying moderate digital governance (e.g., scheduled device breaks, app limiters) to model balanced behavior. These approaches are especially important for adolescents in high-risk fsQCA profiles (e.g., NS1), where both SC and HA are compromised. In such cases, adolescent self-regulation must be supported through temporary external structure until internal regulation becomes viable. Evidence from the SEM model underscores that enhancing SC and TM through family-level guidance may buffer the indirect impact of MPD on exercise.

### 5.3 School-level strategies: building behavioral and motivational competencies

Schools are uniquely positioned to integrate behavioral, educational, and environmental levers. At the behavioral level, physical education curricula could embed brief goal-setting and self-regulation activities, enabling students to plan around digital distractions and anticipate motivational lapses. Time management and prioritization skills may also be included in advisory programs or co-curricular workshops. At the motivational level, lessons in health education should activate personal agency by linking physical activity to outcomes salient to adolescents (e.g., mood, academic performance, peer interaction). Given that HA emerged as the strongest mediator, fostering health beliefs within school settings is crucial. Environmental strategies such as “phone-free” zones during exercise periods or gamified campus fitness challenges can reinforce behavioral norms. These measures serve not only general prevention goals but can also respond to the diversity uncovered in fsQCA: some students require more intensive structuring, while others benefit from digital reframing strategies, such as using fitness apps or wearable trackers to transform the smartphone from distraction to motivator.

### 5.4 Policy-level implications: reshaping ecological conditions

At the macro level, policy interventions can shape the broader digital-health environment in which adolescents operate. Public health messaging campaigns should focus on heightening the perceived relevance of exercise and communicating the cumulative health risks of sedentary screen behavior. Such campaigns may utilize youth-centric platforms (e.g., TikTok, short-video apps) and adopt language that emphasizes autonomy and aspiration. In parallel, education policy can support digital hygiene training in school curricula, promote physical activity benchmarks as academic performance enhancers, and fund infrastructure that facilitates movement-rich environments in communities. From a technological standpoint, partnerships with app developers can promote the creation and dissemination of engaging, evidence-based movement applications for adolescents. These interventions complement individual-level change by reengineering external conditions to favor active lifestyles.

### 5.5 Toward integrated, configuration-sensitive strategies

Finally, the fsQCA findings call attention to the importance of tailoring intervention content and intensity based on adolescents’ psychological profiles. For adolescents with relatively preserved health beliefs but time disorganization (e.g., Path NS3), light-touch time-planning supports may suffice. Conversely, for the most vulnerable profiles (e.g., NS1), multifaceted interventions combining digital behavior restructuring, motivational activation, and adult scaffolding are needed. In all cases, health awareness emerged as a consistent ingredient in high-activity profiles, underscoring its centrality as a universal intervention target. Future programs may benefit from developing modular components that can be flexibly assembled based on adolescents’ specific configuration of needs and strengths.

In sum, effective interventions must be layered, theory-informed, and configuration-sensitive. The combined insights of SEM and fsQCA highlight that reducing mobile phone dependency is necessary but insufficient; lasting impact requires simultaneous investment in self-regulation, temporal planning, and motivational alignment—delivered through coordinated actions across home, school, and policy systems.

## 6 Limitations and future directions

While this study offers novel insights into the behavioral mechanisms linking mobile phone dependency (MPD) and adolescent exercise participation (AEP), several limitations should be acknowledged. First, the cross-sectional design precludes strong causal inferences. Although we interpreted the model structure based on prior theory, bidirectional or reverse causality remains possible—for instance, lower physical activity could lead to increased phone use, or high self-control might simultaneously reduce MPD and promote exercise. Future studies should adopt longitudinal or experimental designs to verify temporal ordering, ideally through interventions that modify smartphone usage or enhance self-regulatory capacities and assess downstream changes in exercise behavior.

Second, the reliance on self-report data may introduce common method bias and response inaccuracy. Although statistical diagnostics showed no major threat, behaviors such as screen time, self-control, or physical activity are prone to recall errors and social desirability effects. Future work should incorporate objective measures (e.g., wearable trackers, app-based usage data) and multi-informant reports to improve construct validity and capture nuanced patterns such as exercise intensity or digital multitasking.

Third, the generalizability of findings is limited by the sample’s sociocultural context (Chinese adolescents grade 5–9). While geographically diverse, the sample reflects a relatively homogeneous digital and educational environment. Replications in other cultural settings and age groups are needed to examine the consistency of mediation and configurational pathways. Of particular interest is whether the compensatory mechanism identified in path S3 (where high internal resources offset high MPD) generalizes across samples. Additionally, future fsQCA studies could incorporate broader variables—such as family support, peer norms, facility access, or mental health—to construct a more comprehensive socio-ecological understanding of youth physical activity in digital environments.

Lastly, this study focused on exercise participation as the primary outcome, without directly examining related domains such as mental health, academic performance, or sleep quality. Future research should explore whether the negative impacts of mobile phone overuse—and the protective roles of self-control, time management, and health awareness—extend to these broader outcomes. Given the well-established benefits of physical activity, it would be valuable to investigate whether enhancing exercise participation also yields improvements across other areas of adolescent functioning. A promising direction lies in developing integrative interventions that jointly reduce digital dependency and promote healthy routines, with outcomes assessed across multiple dimensions of adolescent well-being. In doing so, future studies may also leverage emerging data-driven approaches such as machine learning (Zang et al., 2025) to uncover latent subgroups, optimize intervention targeting, and enhance predictive precision in behavioral research.

## 7 Conclusion

This study employed a dual-method approach, integrating covariance-based structural equation modeling (CB-SEM) and fuzzy-set qualitative comparative analysis (fsQCA), to investigate how mobile phone dependency (MPD) influences adolescent exercise participation (AEP) through self-control, time management, and health awareness. CB-SEM results confirmed that MPD had a significant negative impact on AEP both directly and through multiple mediating pathways, including several chained effects involving the three psychological mechanisms. fsQCA further revealed that high AEP could be achieved via distinct combinations of conditions: in two configurations, low MPD emerged as a core enabling factor, while a third showed that strong internal resources could offset the influence of MPD regardless of its presence. In contrast, non-high AEP was consistently associated with high MPD and a simultaneous lack of self-control, time management, and health awareness. These findings underscore MPD as a pivotal condition shaping adolescent physical activity, operating both as an individual risk factor and as part of broader psychological configurations.

## Data Availability

The original contributions presented in the study are included in the article/Supplementary material, further inquiries can be directed to the corresponding author.
